# Total liquid ventilation in a porcine model of severe acute respiratory distress syndrome using a new generation of liquid ventilator

**DOI:** 10.1186/s40635-025-00799-9

**Published:** 2025-09-16

**Authors:** Naoto Watanabe, Antoine Bois, Fanny Lidouren, Yara Abi Zeid Daou, Ali Jendoubi, Baptiste Gaborieau, Mathéo Richard, Mathieu Nadeau, Fabrice Paublant, Mickaël Libardi, Sandrine Perrotto, Bijan Ghaleh, Etienne Fortin-Pellerin, Philippe Micheau, Patrick Bruneval, Matthias Kohlhauer, Jean-Damien Ricard, Renaud Tissier

**Affiliations:** 1 Univ Paris Est Créteil, INSERM, IMRB, 94010 Créteil, France; 2 Ecole Nationale Vétérinaire d’Alfort, IMRB, AfterROSC Network, 7 Avenue du Général de Gaulle, 94700 Maisons-Alfort, France; 3 Orixha, 94700 Maisons-Alfort, France; 4 Université Paris Cité, AP-HP, Hôpital Louis Mourier, DMU ESPRIT, Service de Médecine Intensive Réanimation, 92700 Colombes, France; 5 Université Paris Cité, INSERM UMR1137, IAME, 75018 Paris, France; 6 Pediatric Department, Université de Sherbrooke, Sherbrooke, Canada; 7 Department of Mechanical Engineering, Faculty of Engineering, Université de Sherbrooke, Sherbrooke, Canada; 8 Service d’Anatomie Pathologie, Hôpital Européen Georges Pompidou, AP-HP, Paris, France

**Keywords:** Mechanical ventilation, Acute respiratory distress syndrome, Liquid ventilation

## Abstract

**Background:**

Total liquid ventilation (TLV) has been experimentally proposed as an alternative treatment for the management of Acute Respiratory Distress Syndrome (ARDS). Recent technological advances have led to the evaluation of a TLV prototype in patients resuscitated after cardiac arrest. Here, our goal was to determine whether a derived version of this prototype, so-called LV4B (*liquid ventilation for breathing*), could be used for normothermic TLV in a swine model of severe ARDS.

**Methods:**

Swine were anesthetized and instrumented for respiratory and hemodynamic evaluation. ARDS was induced by one or two administrations of oleic acid (0.1 mg/kg), until reaching a PaO2/FiO2 ratio < 100 mmHg. After ARDS induction, animals were allocated to undergo 60 min of either gas ventilation continuation (Control group) or TLV using a prototype that continuously controls respiratory rate (RR), liquid tidal volume (LqVt) and end-expiratory liquid volume (EELqV, respectively). Perfluorooctyl bromide was used as breathable liquid.

**Results:**

After ARDS induction and group allocation, 2/5 animals (40%) survived in the Control groups versus 5/5 in the TLV group (100%). In the Control group, premature deaths were related to sustained hypoxemia (PaO_2_ < 50 mmHg) with hemodynamic failure. Surviving animals presented a trend toward better oxygenation in TLV versus Control, without achieving statistical significance due to the low number of survivors in the Control group. PaCO_2_, blood pH, lactate levels, or pulmonary and systemic hemodynamics were not different between groups in survivors. In the TLV group, the average LqVt, EELqV, and respiratory rate (RR) were 12.6 ± 0.4 mL/kg, 22.9 ± 2.9 mL/kg, and 5.3 ± 0.5 breath/min (mean ± SEM) at the end of the procedure, respectively. In all animals, pulmonary debris were washed out from the lung and collected by the TLV device throughout the procedure. After necropsy, histopathological examination demonstrated a significantly lower extent of inflammatory and congestion lesions in TLV versus Control.

**Conclusions:**

TLV with a liquid ventilator controlling EELqV, RR and LqVt is feasible and safe in large animals in a severe model of ARDS. This opens promising perspectives and warrants further investigation, including prolonged treatment durations and long-term follow-up.

**Supplementary Information:**

The online version contains supplementary material available at 10.1186/s40635-025-00799-9.

## Background

Acute respiratory distress syndrome (ARDS) represents a persistent public health issue, affecting more than 10% of critically ill patients [1, 2]. Despite dedicated organ support through protective mechanical ventilation or extracorporeal membrane oxygenation (ECMO), the risk of complications is extremely high, and the overall mortality rate reaches 30–50% [1]. Thus, there is an unmet medical need for alternative approaches, particularly in patients with strong inflammatory responses and dramatically reduced lung compliance.

Total liquid ventilation (TLV) with organofluorine liquids has been proposed as a possible alternative for severe ARDS treatment [3]. The physicochemical characteristics of these breathable liquids, including high solubility for O_2_ and CO_2_, low surface tension, and anti-inflammatory effect, could improve gas exchanges and promote lung recovery as compared to the conventional approaches [3, 4]. TLV is radically different from partial liquid ventilation (PLV), which consists of conventional gas ventilation after lung filling with breathable liquids. This latter approach failed to demonstrate clinical benefits and was associated with an increased risk of pneumothorax in large-scale clinical studies, likely due to the use of high tidal volumes (Vt, typically > 8 mL/kg) in lungs already distended by the liquid [5]. In comparison, TLV requires a dedicated device but provides actual tidal ventilation with liquids and completely abolishes the air–liquid interface as compared to PLV. To the best of our knowledge, this technique has not been evaluated in patients until now, due to the lack of a dedicated medical device. Promising results were observed with improved oxygenation and reduced lung injury in small animals [6–8], as well as in sheep undergoing up to 24 h of TLV after ARDS induction as compared to gas ventilation [8, 9]. In the latter studies, oxygenation was improved, but the TLV prototype did not allow continuous and primary control of the end-expiratory liquid volume (EELqV) throughout the procedure, whereas such control is critical to prevent the risk of lung trauma. When EELqV is controlled at a low level, this also allows for an increase in the liquid Vt (LqVt) to improve gas exchange, since the risk of lung trauma is actually determined by the end-inspiratory liquid volume as a sum of Vt and EELqV. Recently, a new device for TLV (*Vent2Cool*^®^) was developed for body cooling after cardiac arrest, and a clinical study is now ongoing to demonstrate the safety of this technology in resuscitated patients [10]. Vent2Cool precisely controls tidal volumes to maintain end-expiratory lung liquid volume and incorporates strategies to limit airway collapse during the active expiration phase of TLV. The latter aspect was shown to be a challenge during TLV [11]. For the present investigation, Vent2Cool technology was adapted to perform normothermic TLV for ARDS management. Using the same engineering principles, we evaluated a new prototype, so-called LV4B (liquid ventilation for breathing), in a swine model of severe ARDS. We hypothesized that LV4B would enhance oxygenation and short-term survival in a large animal ARDS model while accurately estimating and controlling pulmonary liquid volume during a 1-h TLV and implementing automated strategies to limit airway collapse during expiration.

## Methods

### Animal preparation

Twelve female Large White pigs, weighing 33 ± 2 kg, were sedated by a mixture of zolazepam and tiletamine (10 mg/kg, i.m.), followed by propofol (1 mg/kg i.v.). Analgesia was applied by methadone (0.3 mg/kg i.m.). Anesthesia was maintained by the continuous administration of propofol (10 mg/kg/h i.v.). After endotracheal intubation with a 7.0 mm cuffed endotracheal tube, animals were submitted to conventional mechanical ventilation (Viasys Avea^®^; Vt = 6 mL/kg; inhaled fraction of oxygen [FiO_2_] = 30%; respiratory rate [RR] = 30 ± 5 breaths/min; positive end-expiratory pressure [PEEP] = 5 cmH_2_O) in the supine position. RR was modified when needed to maintain normocapnia and normoxia. Sheaths were inserted into the femoral arteries for invasive blood pressure. A Swan-Ganz pulmonary artery catheter (Edwards Lifescience, Irvine, USA) was inserted through the right external jugular vein for the measurement of pulmonary arterial, right atrial, and pulmonary wedge pressures. A central venous catheter was also inserted for drug administration.

### Lung injury

After stabilization and baseline measurements, ARDS was induced by the administration of oleic acid (C_18_H_34_O_2_; Sigma-Aldrich; St. Louis, Missouri) into the right atrium. The criterion for severe ARDS was defined as arterial blood partial pressure of O_2_ (PaO_2_)/FiO_2_ ratio < 100 mmHg, as previously described for large animal models of ARDS [8, 12]. A first infusion of oleic acid was delivered over 20 min at the dose of 0.1 mL/kg diluted into 20 mL of autologous whole blood and 5000 UI of unfractionated heparin. Twenty minutes after the end of the infusion, the PaO_2_/FiO_2_ ratio was evaluated. If it was superior to 100 mmHg, another administration of oleic acid was conducted over 20 min, followed by another 20 min before new arterial blood gas evaluation. If the ratio was inferior to 100 mmHg after the first oleic acid administration, animals received only whole blood with unfractionated heparin instead of oleic acid. In case of systemic hypotension during oleic acid administration, norepinephrine was applied as needed to target a mean arterial pressure (MAP) at 65 ± 5 mmHg. During the ARDS induction, animals were ventilated using the same parameters as baseline. Continuous monitoring of SpO_2_ was used as an indicator of lung injury and FiO_2_ was progressively increased in order to target oxygenation saturation > 94%.

### Experimental treatments after the initial lung injury

At the end of the 80 min required for ARDS induction, animals were paralyzed with rocuronium (3 mg/kg i.v., followed by 1 mg/kg/h i.v.). Then, they were assigned to either conventional mechanical ventilation (Control group) or TLV (TLV group) by blocked randomization, respectively. In the Control group, animals were maintained under conventional mechanical ventilation (AGV02430 Viasys^®^; Vt = 6 mL/kg; RR = 30 ± 5 breaths/min; positive end-expiratory pressure [PEEP] = 5 cmH_2_O; FiO_2_ = 100%) for 60 min. After 60 min, animals were euthanized and lungs were sampled for further analyses. Since animals were deeply anesthetized, animals were not euthanized prematurely, even in case of severe hypoxemia or hypotension ultimately leading to cardiac arrest and premature death.

**Fig. 1:**
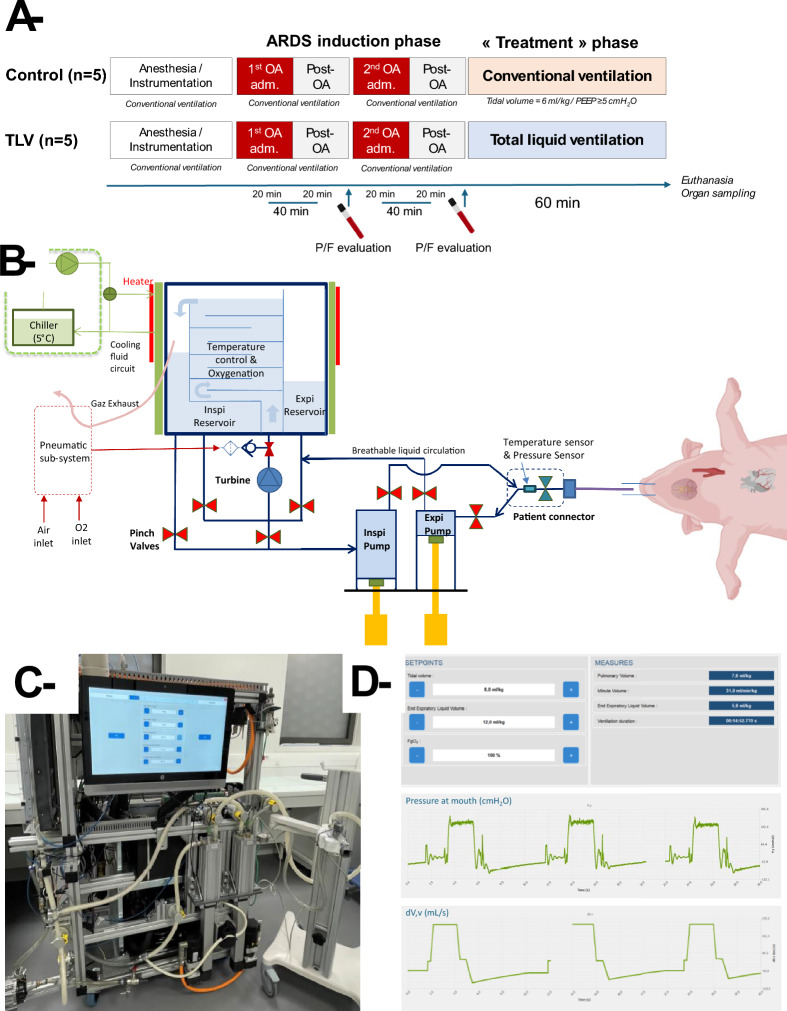
**Panel A**, Schematic representation of the experimental design of the study; **Panel B**, Schematic representation of the LV4B prototype of liquid ventilator; **Panel C**, Picture of the LV4B prototype; **Panel D**, Different screens of the graphic user interface of LV4B prototype. *LV4B* liquid ventilation four breathing; *OA* oleic acid; *TLV* total liquid ventilation

In the TLV group, animals were switched from conventional mechanical ventilation to TLV for 60 min. Figure 1 illustrates the LV4B liquid ventilator. This ventilator includes two independent software-controlled piston pumps to insert and withdraw the liquid in the lung, a turbine, and seven software-controlled synchronized pinch valves to guide the liquid flow in the ventilator (Fig. 1b). A patient connector equipped with temperature and pressure sensors allows the connection of LV4B to the endotracheal tube. During TLV, a technical bag receives the expired liquid where the breathable liquid is oxygenated and CO_2_ scrubbed by bubbling a controlled mix of medical air and pure O_2_ through a gas injector. The O_2_ concentration in the liquid is controlled by modifying the fraction of O_2_ administered into the gas injector (FgO2). Temperature is regulated by an aluminum heat exchanger, composed of heaters glued on, and cooling fluid circulating in aluminum plates sandwiching the technical bag. Once the endotracheal tube was connected to LV4B, the lungs were slowly filled by LV4B with oxygenated and temperature-controlled perfluorooctyl bromide (PFOB, Exfluor, Austin Texas, USA). This slow filling takes place by 3 liquid cycles with increasing respiratory rates and expiratory Vt, from 3.5 to 6/min and 4–6 mL/kg, respectively. This phase allows to evacuate most of the remaining gas from the lung (degassing phase). TLV was then initiated with the following parameters: LqVt = 8 mL/kg, EELqV = 12 mL/kg, RR = 6/min, expiratory flow time constant = 8 s, and fraction of oxygen in the liquid treatment unit (FgO_2_) = 100%. Supplemental Fig. 1 illustrates a schematic representation of lung liquid volume evolution during TLV. Breathable liquid temperature was set to maintain normothermia (38 °C in swine) by controlling expired breathable liquid temperature at the patient connector using the previously described LV4B heat exchanger. LV4B measured end-inspiratory liquid volume (EILqV) and EELqV using load cells that continuously monitor changes in the mass of the ventilatory liquid. By precisely knowing the initial primary volume introduced during device preparation, LV4B estimates EILqV and EELqV at the end of inspiration and expiration for each cycle, respectively. The quality of this estimation has been experimentally validated (Supplemental Fig. 2). LqVt and RR were modified to optimize oxygenation or ventilation.

Throughout the procedure, we evaluated at the patient connector the dynamic airway pressure wave forms at expiration, which evidenced negative levels due to active expiration (Fig. 1d and Supplemental 3). In case of excessive reduction of airway pressure at the end of expiration, the EELqV set-point was increased as such depression is a marker of insufficient lung filling. LV4B also automatically modified expiratory flow profiles in a real-time manner to prevent putative airway collapse during active expiration. As illustrated in Supplemental Fig. 3, when airway pressure dropped below the threshold at each moment of the expiratory phase, the target expiratory flow was automatically reduced by LV4B and expiration was prolonged. The percentage of flow reduction was initially set to 20% in LV4B. As explained in the results section, this percentage was modified to 50% in the last two animals of the TLV group.

After 60 min of follow-up, we ended the procedure and drained lung liquid to the maximum extent with a pressure-controlled forced expiration. The liquid was collected, and animals were immediately euthanized and lungs were excised for further analysis.

### Lung sample analysis

Four samples were harvested from the different parts of the diaphragmatic lobes (left / right and ventral / dorsal) and stored at −80 °C for biochemical analysis or fixed in formaldehyde for pathology (4%). Fixed samples were prepared for histopathological analysis and stained with hematoxylin–eosin–saffron. Interstitial and alveolar inflammatory lesions, hemorrhage, congestion, and edema were blindly semi-quantified on a 0 (normal lung) to 5 (extensive and generalized lesion) scale, as previously described [6]. Protein extraction from the frozen samples collected from the ventral and dorsal lung regions was performed using a cell lysis kit (Bio-Plex^®^, Bio-Rad, Hercules, California). Total protein and inflammatory cytokines (IL-1β, IL-6) were quantified by advanced protein assay and enzyme-linked immunosorbent assay (ELISA), respectively.

### Statistical analysis

Statistical analyses were conducted with GraphPad Prism (GraphPad Software, California, USA). Continuous variables were compared among experimental groups using a mixed effects analysis for repeated measures considering “time,” “group,” and “time x group” effects. Post-hoc analyses were performed for group comparisons using a Holm–Šídák analysis. Values were not compared among time points for repeated measures comparisons. Survival was compared between groups using a log-rank test. Data were expressed as individual values and mean ± SEM (standard error of the mean). *P* values < 0.05 were considered significant.

## Results

Twelve animals were enrolled in the present study, among which ten animals achieved the inclusion criteria (PaO_2_/FiO_2_ < 100 mmHg) after the administration of 1 (*n* = 2 and 1 in TLV and Control group, respectively) or 2 (*n* = 3 and 4 in TLV and Control group, respectively) doses of oleic acid, respectively. The two other animals either died prematurely (*n* = 1) during the administration of oleic acid or did not present a PaO_2_/FiO_2_ < 100 mmHg after administration of oleic acid (*n* = 1). Finally, ten animals were allocated to the two different groups after achievement of the inclusion criteria (*n* = 5 per group).

### Short-term survival after ARDS induction

**Fig. 2:**
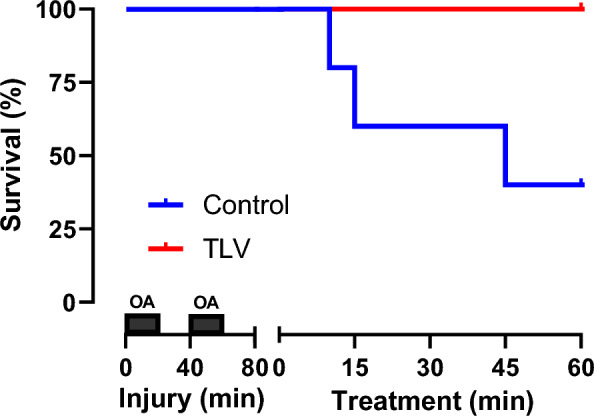
Survival in the different groups following the induction phase of the acute respiratory distress with oleic acid (OA) administration. *n* = *5 in both groups; TLV, total liquid ventilation*

In the Control group, 3 out of 5 animals died from refractory hypoxemia and hemodynamic collapse at 10, 15, and 45 min of follow-up, respectively. In the TLV group, all animals survived throughout the entire 60 min follow-up period, achieving a 100% survival rate versus 40% in the Control group (Fig. 2).

### Evaluation of the specific parameters of TLV

**Fig. 3:**
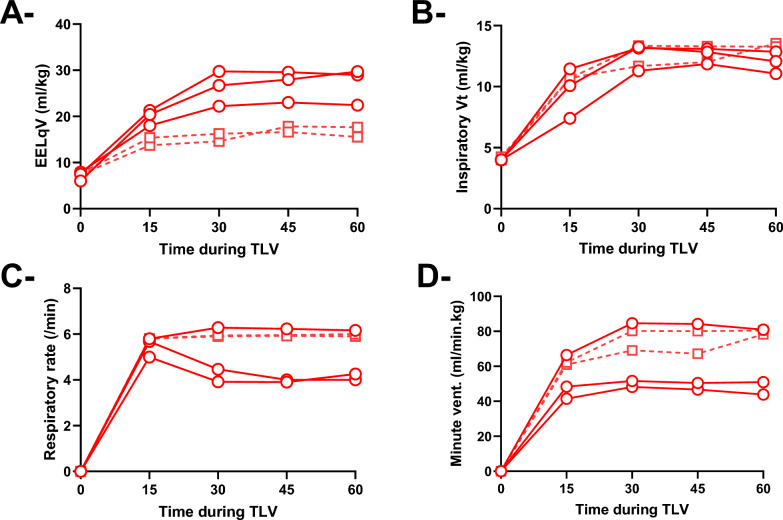
Measured values of end-expiratory liquid volume (EELqV, Panel A), inspiratory liquid tidal volume (Vt, Panel B), respiratory rate (RR, Panel C) and minute ventilation (Panel D) during total liquid ventilation (TLV) in each animal of the corresponding group. Circles and squares represent the individual values obtained during TLV with either a 20% (*n* = 3) or 50% (*n* = 2) reduction of the expiratory flow in case of expiratory flow limitations, respectively. With the second one, respiratory rate and EELqV were maintained at higher and lower levels, respectively. This was associated with greater minute ventilation with 50% of expiratory flow reduction in case of flow limitation as compared to 20%. *Data are represented as individual values*

At the beginning of the TLV procedure, we observed exudates and hemorrhagic debris being washed out of the lung. As illustrated in Fig. 3, LqVt, EELqV, and RR were set within the ranges of 8–14 mL/kg, 12–30 mL/kg, and 4–6 breaths/min, respectively, throughout the procedure. These parameters were modified depending upon the results of arterial blood gas analysis measured every 15 min. EELqV was progressively increased during the first 30 min of TLV in order to optimize gas exchanges and, if necessary when automatic flow reduction was inefficient, to prevent airway collapse at expiration. It was maintained below 30 mL/kg in all animals.

In the first three animals submitted to TLV, the flow reduction applied at expiration to prevent airway collapse was set to 20% in LV4B (Circles, Fig. 3). This was not sufficient to fully prevent unexpected airway depression during expiration in two animals, requiring a decrease in RR and an increase EELqV to control the hazard of expiratory airway collapse.

In the last two animals submitted to TLV, the percentage of flow reduction in such situations was increased to 50% in LV4B (Squares, Fig. 3). This was sufficient to automatically prevent the hazard of airway collapse due to active expiration and airway depression, with minimal impact on the adjusted expiratory time and RR. It was then not required to modify RR, thus maintaining minute ventilation and EELqV to manage expiratory profile. Those parameters could be maintained at higher and lower levels, respectively.

### Arterial blood gases, respiratory function and hemodynamics

**Fig. 4:**
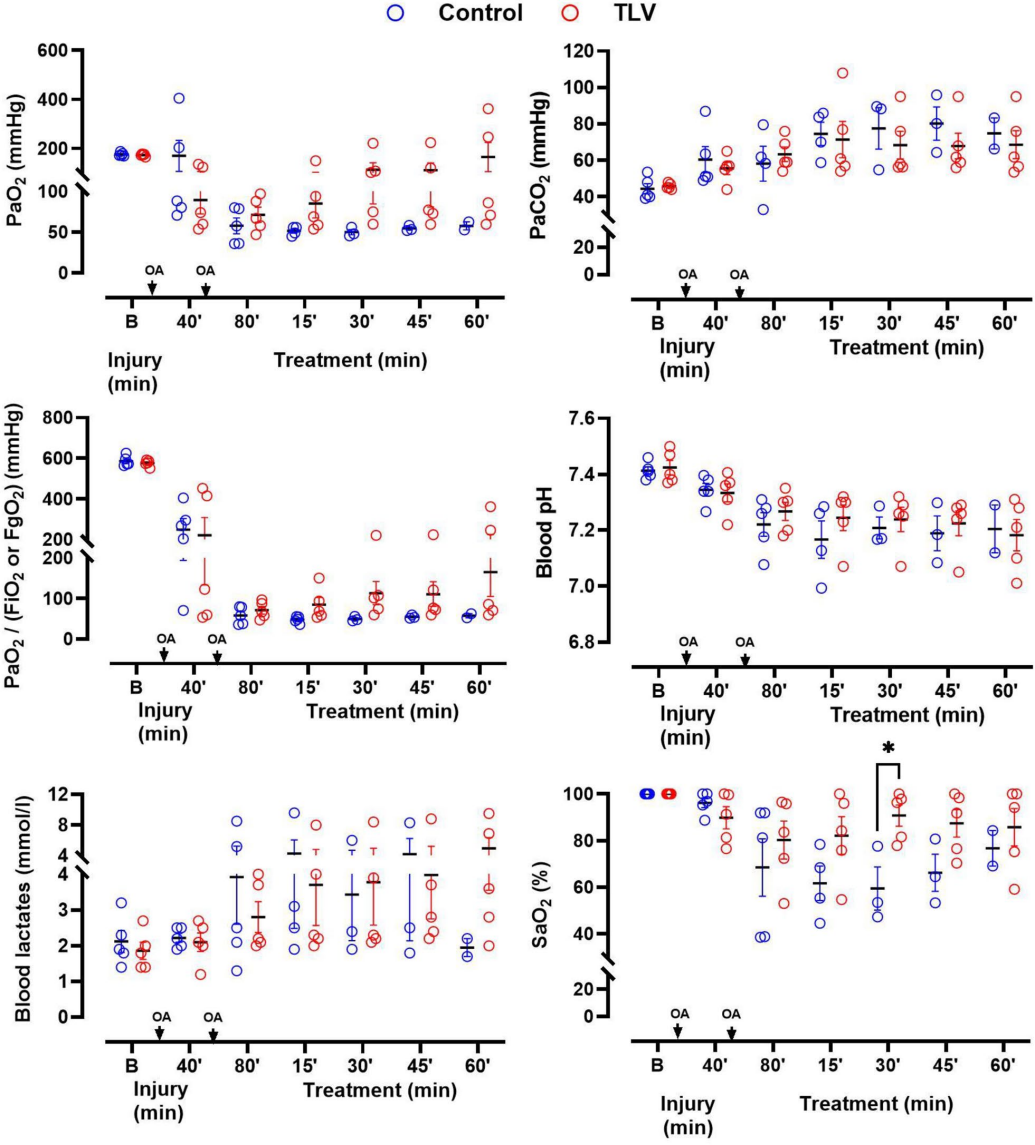
Blood gases, pH and lactate levels in the surviving animals from the different groups following the induction phase of the acute respiratory distress with oleic acid (OA) administration. *Data are represented as individual values of surviving animals and mean* ± *S.E.M.; PaO*_*2*_*, arterial blood partial pressure of O*_*2*_*; PaCO*_*2*_*, arterial blood partial pressure of CO*_*2*_*; FiO*_*2*_*, inhaled fraction of oxygen; FgO2, fraction of oxygen administered into the breathable liquid of the LV4B prototype; SaO*_*2*_, arterial blood oxygen saturation; *, p < *0.05 versus Control*

**Fig. 5:**
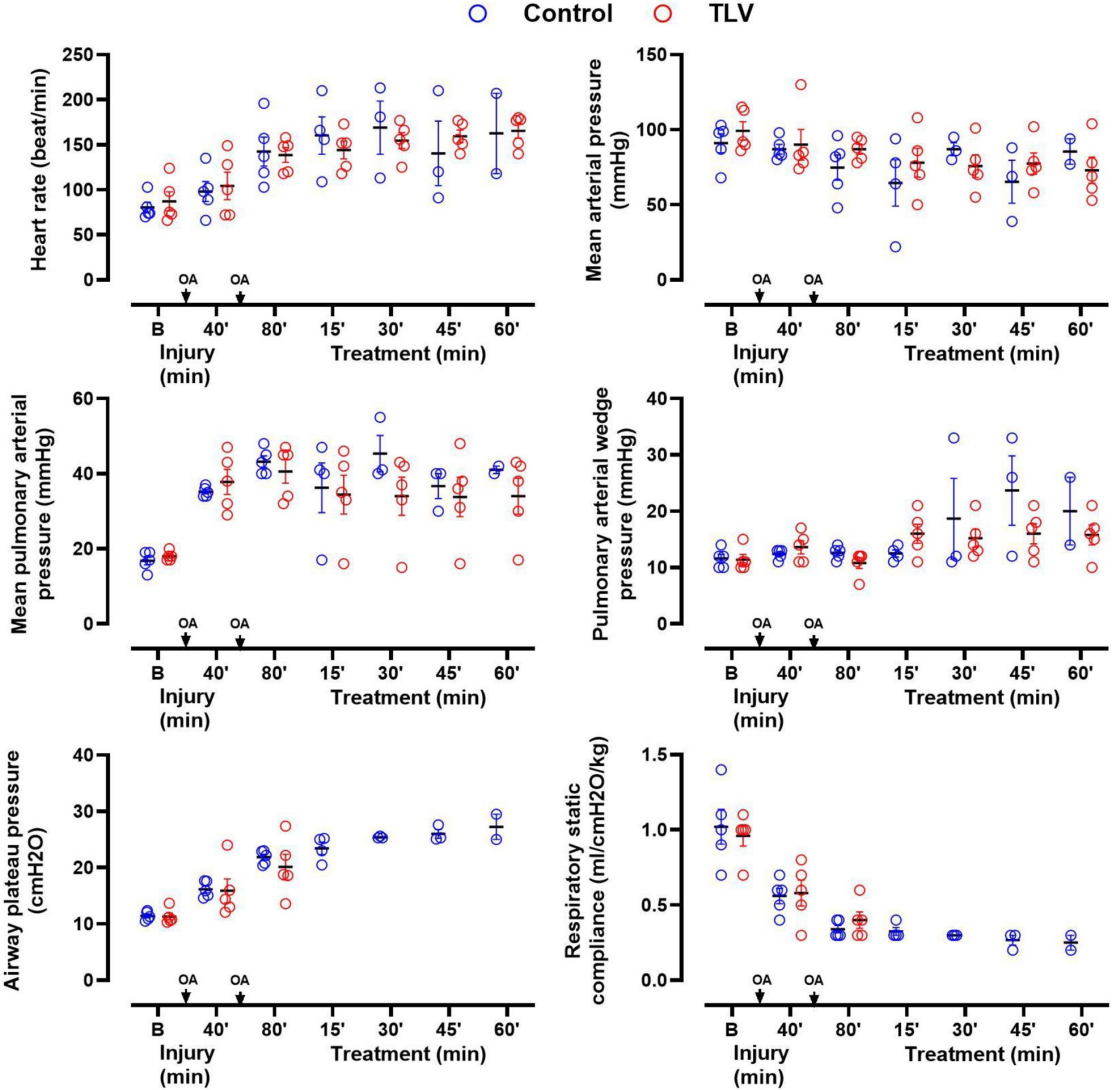
Hemodynamic and respiratory parameters of the surviving animals in the different groups following the induction phase of the acute respiratory distress with oleic acid (OA) administration. *Data are represented as individual values of surviving animals and mean* ±S.E.M.; TLV* total liquid ventilation; respiratory parameters cannot be measured during TLV in the corresponding group*

As illustrated in Figs. 4 and 5, hemodynamic parameters, arterial blood gases, and respiratory compliance were not recovering throughout the follow-up in the surviving animals of the Control group. Blood gases, lactate levels, and pH were also not significantly different among groups at baseline or after ARDS induction in surviving animals. However, a trend toward better oxygenation was observed in TLV versus Control. Indeed, 3 Control animals showed PaO_2_ values below 50 mmHg (at FiO_2_ = 100%), leading to refractory shock and premature death. In comparison, no animal exhibited PaO_2_ values below 50 mmHg in the TLV group.

Heart rate, mean systemic and pulmonary arterial blood pressure, and pulmonary arterial wedge pressure were not significantly different among groups (Fig. 5). Airway plateau pressure and respiratory static compliance were also not different between groups until the start of TLV, after which they could not be evaluated in the TLV group due to liquid ventilation. As stated above, three animals presented hemodynamic collapses with MAP falling below 50 mmHg despite norepinephrine administration in the Control group.

### TLV attenuated lung lesions at histology but led to increased cytokine concentrations

**Fig. 6:**
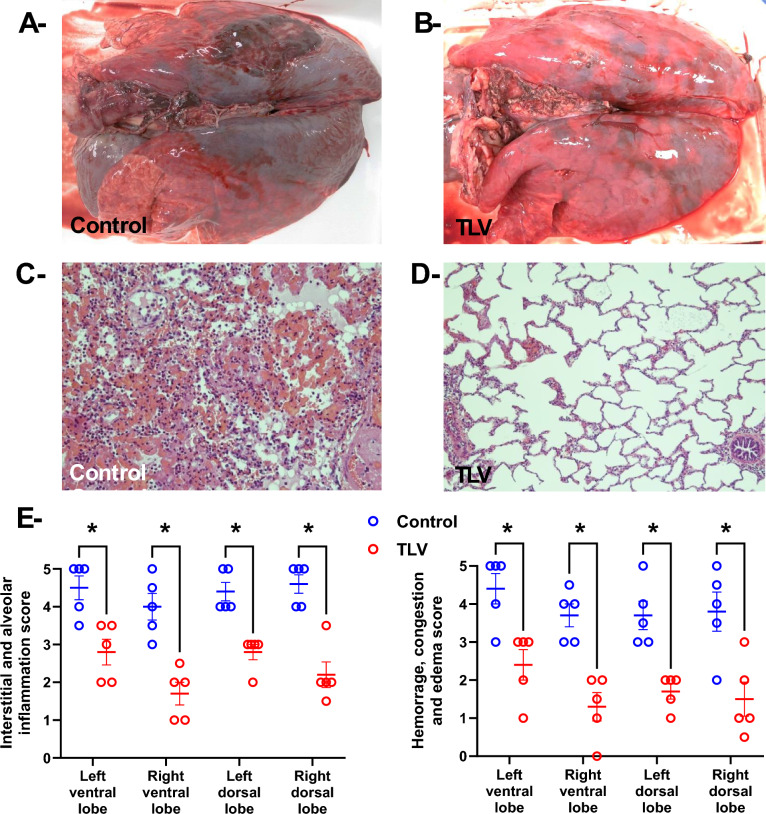
Panels A and B, Gross morphological appearance of the lung in a representative animal of the Control and TLV groups, respectively. Panels C and D, Histological appearance of the lung in a representative animal of the Control and TLV groups, respectively. Hyaline membrane can be seen on the Control samples with extensive congestion, edema, and infiltration by inflammatory cells. Panel E, Individual scores of inflammation (left panel) or congestion (right panel) in the lung lobes of all animals from Control and TLV groups. *, p < *0.01 versus Control group; Data are represented as individual values and mean* ±S.E.M.

The pathological investigation was conducted in all animals submitted to TLV or Control procedure, even those prematurely deceased before the end of the 60 min treatment phase. As illustrated in Fig. 6, the gross examination of the lung after excision evidenced a more severe hemorrhagic appearance in the lung from the Control versus TLV group. The blind histological examination confirmed this phenomenon with reduced scores of hemorrhage, congestion, and edema in TLV versus Control. Importantly, scores of interstitial and alveolar inflammation were also significantly reduced in TLV versus Control. The biochemical evaluation of the IL-6 and IL-1β concentrations in lung samples demonstrated higher concentration in TLV versus control lungs (2439 ± 137 vs. 345 ± 11 pg/mg of protein, *p* < 0.05).

## Discussion

In the present study, we demonstrated that TLV with the LV4B prototype is feasible in a swine model of severe ARDS leading to high mortality in control conditions. This prototype was designed to monitor and control pulmonary liquid volumes during a short course of TLV. After one hour of follow-up, TLV was well tolerated in terms of hemodynamic and systemic responses in animals with severe ARDS. TLV also tended to prevent the appearance of severe hypoxemia and subsequent mortality from hemodynamic collapse, while also reducing lung lesions at histology.

Importantly, we used an oleic acid model of ARDS, which causes alveolar membrane damage and “*reproduce most of the pathophysiology of clinical [..] ARDS*” [12]. This model reliably produces lung injury with rapid and severe hypoxemia, pulmonary hypertension, and respiratory compliance decrease [12]. It is considered particularly relevant to study severe ARDS complicated by acute heart failure or pulmonary hypertension, while less suitable for studying the inflammatory pathophysiology of ARDS, as compared to other models.

In previous studies, the benefits of TLV have been documented in different ARDS models including meconial aspiration in lambs [13], post-cardiac arrest lung injury in piglets [14] and oleic acid in sheep [8, 9]. In the latter large animal studies, TLV was maintained during 4 [9] and 24 h [8], respectively, demonstrating the feasibility of a prolonged period of TLV. In the present study, animals were evaluated during only one hour after the initial injury since our goal was to evaluate the performance of a new generation of liquid ventilator capable of controlling EELqV and optimizing expiratory flow through a dedicated algorithm designed to prevent airway collapse. Airway collapse management is one of the biggest challenges during TLV due to active expiration [11, 15]. Failure to do so impairs proper ventilation and/or necessitates increasing lung filling with a risk of subsequent lung trauma.

Importantly, EELqV level appeared relatively low with the LV4B prototype as compared to the initial lung filling observed in previous TLV studies [8, 9]. For instance, the lung was initially filled with 30 mL/kg of breathable liquid in the long-term TLV study in sheep [8]. This level corresponds to the upper range observed in our study and was only reached in 2/5 animals. In addition, this level was only progressively achieved in the present study, whereas previous studies [8, 9] started at this level with a manual infusion into the endotracheal tube before connection to the TLV prototype. In comparison, LV4B performed a reproducible, programmed lung filling to facilitate TLV implementation in clinical conditions. This filling phase is known to be at risk since gas elimination should be concomitant with the progressive liquid administration to prevent lung trauma [3, 5]. If not properly managed, overdistention of the lung might occur. Therefore, LV4B’s ability to fill the lung with real-time monitoring of pulmonary liquid volume brings a significant advancement.

In a previous study in piglets with intact lungs, we have already shown that EELqV should be maintained at low or moderate levels, below functional residual capacity, during short episodes of liquid ventilation [16]. In the middle of a 30 min of TLV, air was indeed still observed in the upper pulmonary territory in this study [16]. Given these findings, the actual lung volume refers to the total volume consisting of both trapped air and actual EELqV. Accordingly, EELqV likely needs to be gradually increased over the first 30 min of the TLV to compensate for the slow elimination of gases, aiming to achieve an optimal filling and avoid the occurrence of airway collapse at end-expiration. The critical pressure promoting airway pressure indeed depends upon the lung filling level of the recruited region [5]. As already described, TLV was performed for 60 min in the present study, during which the EELqV had to be gradually increased over the course of TLV. We speculate that this phenomenon is inevitable in response to the progressive removal of trapped gas during the TLV. Even though we had to increase the EELqV up to 30 mL/kg in some animals, it remained lower than the level reported in former studies. In addition, when examining the difference between the 20% and 50% reduction in expiratory flow for collapse prevention, the 50% reduction showed higher minute ventilation (> 60 mL/kg/min) and lower levels of EELqV (< 20 mL/kg), demonstrating a better resolution of collapse occurrence and confirming the possibility of lowest EELqV. In addition, the stability of the EELqV after 30 min of TLV suggests that the lung was recruited and that air was mostly eliminated. In a previous study in piglets submitted to 180 min of TLV, lungs were indeed fully filled after 60 min with no more trapped air in the upper area evaluated by CT scan [17]. In any case, the relevance of maintaining EELqV at the lowest possible level has been well shown during partial liquid ventilation in rats [18], in which the risk due to air trapping was well evidenced [19].

Notably, despite the short duration of the follow-up, we were able to evidence improved oxygenation in surviving animals of the TLV group, as well as reduced inflammatory and congestive lesions at histology. These effects may be partially attributed to the lung lavage properties of TLV, which washed exudates and hemorrhagic debris from the injured lungs. In previous studies, TLV was even proposed for proper lung lavage in specific conditions such as meconial aspiration [13]. The anti-inflammatory and lung-protective effects of TLV can also be related to increased alveolar recruitment and reduced transpulmonary pressure, strains, and ventilation/perfusion mismatch as compared to gas ventilation [3, 4]. Several studies also suggested a direct anti-inflammatory effect of organofluorine due to the suppression of the air–liquid interface in the alveoli [7, 14]. This is not directly supported by the evaluation of the cytokine levels within the injured lungs in our study, as they were significantly increased in TLV versus control animals, which is inconsistent with the histology data. This discrepancy may be partly due to the shorter follow-up in control animals, which died prematurely from refractory hypoxemia and hemodynamic collapse. This could provoke immunoparalysis and altered immune cell reactivity despite increased recruitment in the pulmonary vascular walls and alveoli. Interestingly, a similar increase in pulmonary cytokine levels was observed in a lamb model of extreme prematurity [20]. This phenomenon could be explained by the fact that organofluorine compounds are cleared by phagocytic cells, leading to cytokine secretion [4, 21]. Supporting this hypothesis, safety studies involving the intravenous administration of PFOB demonstrated increased cytokine blood levels, along with mild, reversible flu-like symptoms, with no further consequence on the longer term [22]. Therefore, this could be a normal consequence of breathable liquid elimination but obviously deserves further investigations in the longer term.

Despite the positive findings observed in the present study, several limitations have to be acknowledged, among which the most important ones are the limited number of animals and the short duration of follow-up. In fact, the limited number of animals might have been too small to achieve adequate statistical power to show significant differences for several parameters, especially considering the premature death of several animals in the Control groups. The necessity to confirm results after prolonged duration has already been discussed, even if the primary goal was here to evaluate the feasibility of TLV with LV4B in very severe conditions. The latter point could also be considered as a limitation since short-term mortality was very high in Control conditions, with major and acute pulmonary hypertension and systemic repercussions. It could be important to evaluate findings in different conditions, e.g., milder ARDS or severe ARDS in combination with ECMO. Indeed, it would allow to determine whether TLV should be considered as an alternative or a bridge to ECMO. Finally, one would argue that we did not fully optimize the protective ventilation in the Control group, which would also require further analysis. For example, PEEP was fixed at 5 mmH_2_O in the Control group and no additional rescue maneuvers were allowed, such as airway suctioning, prone positioning, or recruitment maneuvers. A part of TLV potential benefits could clearly be related to debris removal. In addition, respiratory mechanics remain hard to evaluate during TLV for a proper comparison with the Control conditions. Indeed, airway pressure during TLV differs from gas ventilation, under which this depends mostly on respiratory compliance and resistance. During TLV, inertance must be considered due to the high density and acceleration of the liquid filling of airways and endotracheal tube. Consequently, a direct correspondence between pressures (static or dynamic) measured at the mouth cannot directly be performed during TLV, deserving further investigations.

## Conclusion

In conclusion, TLV with the LV4B new prototype, which controls EELqV in a real-time manner, is feasible in large animals and tends to improve short-term survival through a limitation of refractory hypoxemia in a severe model of ARDS. This opens promising perspectives and deserves further investigations with a prolonged treatment phase and a greater number of animals. Evaluation in other conditions, including milder ARDS conditions or combination with ECMO, might also be important to understand the possible place for TLV in ARDS management. In addition, further validation studies will be necessary to demonstrate the accuracy of EELqV monitoring for prolonged TLV durations of several hours or days. It is also important to state that TLV is not approved for medical use in ARDS and still requires experimental and technological investigations.

## Supplementary Information

Additional file 1.

## Data Availability

Data will be available upon request to the corresponding author.
