# Supplementary material for: Total liquid ventilation in a porcine model of severe acute respiratory distress syndrome using a new generation of liquid ventilator
Source: Intensive Care Med Exp. 2025 Sep 16;13:95. doi: 10.1186/s40635-025-00799-9 (PMC12436666; doi:10.1186/s40635-025-00799-9)
Supplement: Supplementary file 1 — Additional file 1. [file 40635_2025_799_MOESM1_ESM.pdf]

Supplemental Figure 1: Illustration of the evolution of the liquid volume of the lung during two typical cycles of total liquid ventilation with end-expiratory liquid volume (EELqV), with end-inspiratory liquid volume (EILqV) and liquid tidal volume (LqVt).

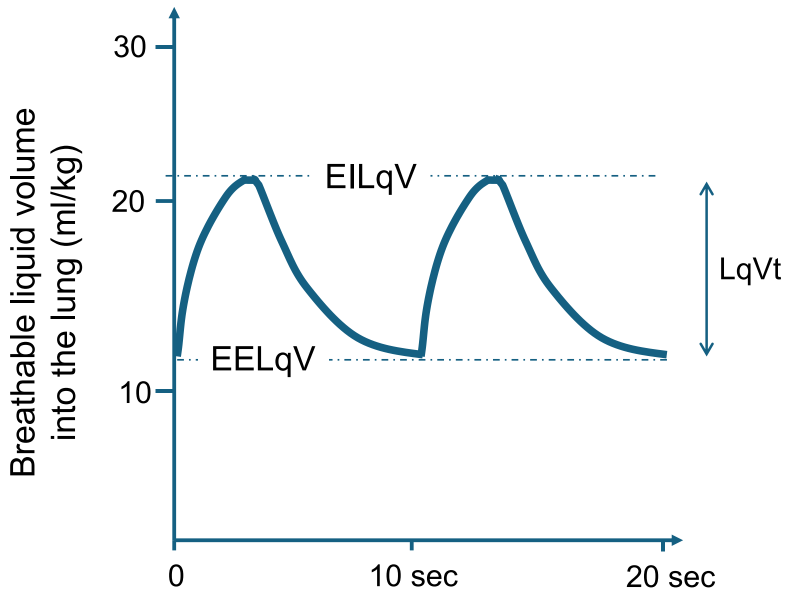

Supplemental Figure 2: Continuous follow-up of breathable liquid volume into the lung in a swine during total liquid ventilation. The target end-expiratory liquid volume (EELqV) set by the clinician is displayed in blue. The liquid volume measured by LV4B is shown in green in a real-time manner. The actual liquid volume calculated by mass measurement using a scale under the animal is shown in red.

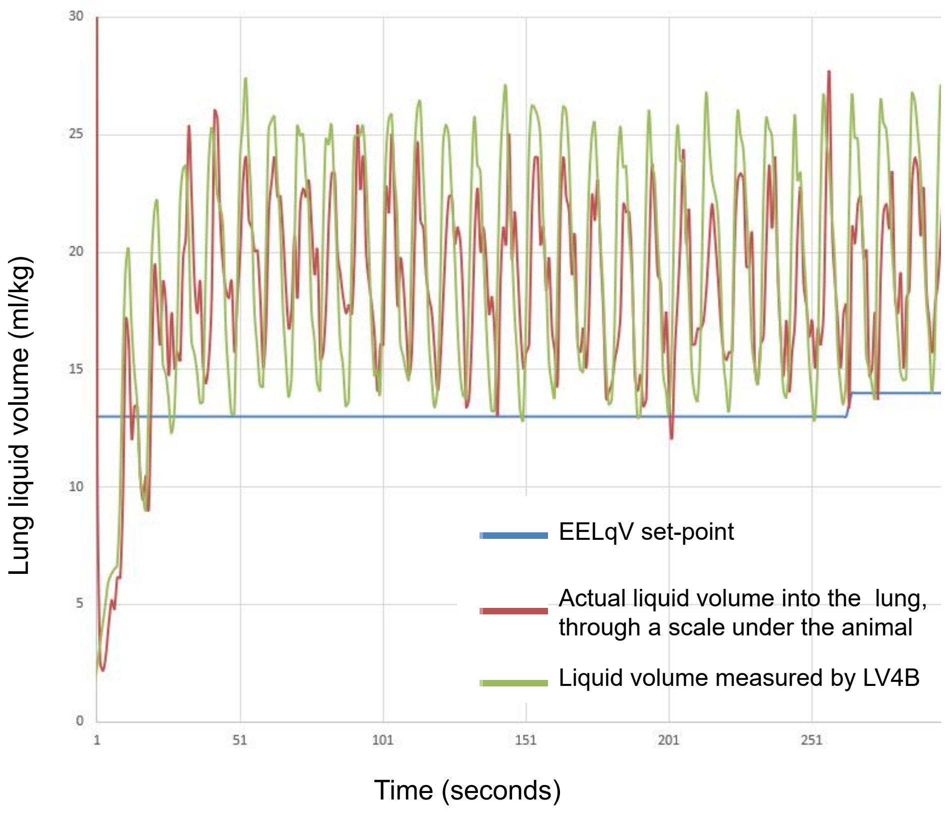

Supplemental Figure 3: Illustration of the principle of expiratory flow order reduction in case of flow limitation evidenced by measured pressure below expected values.

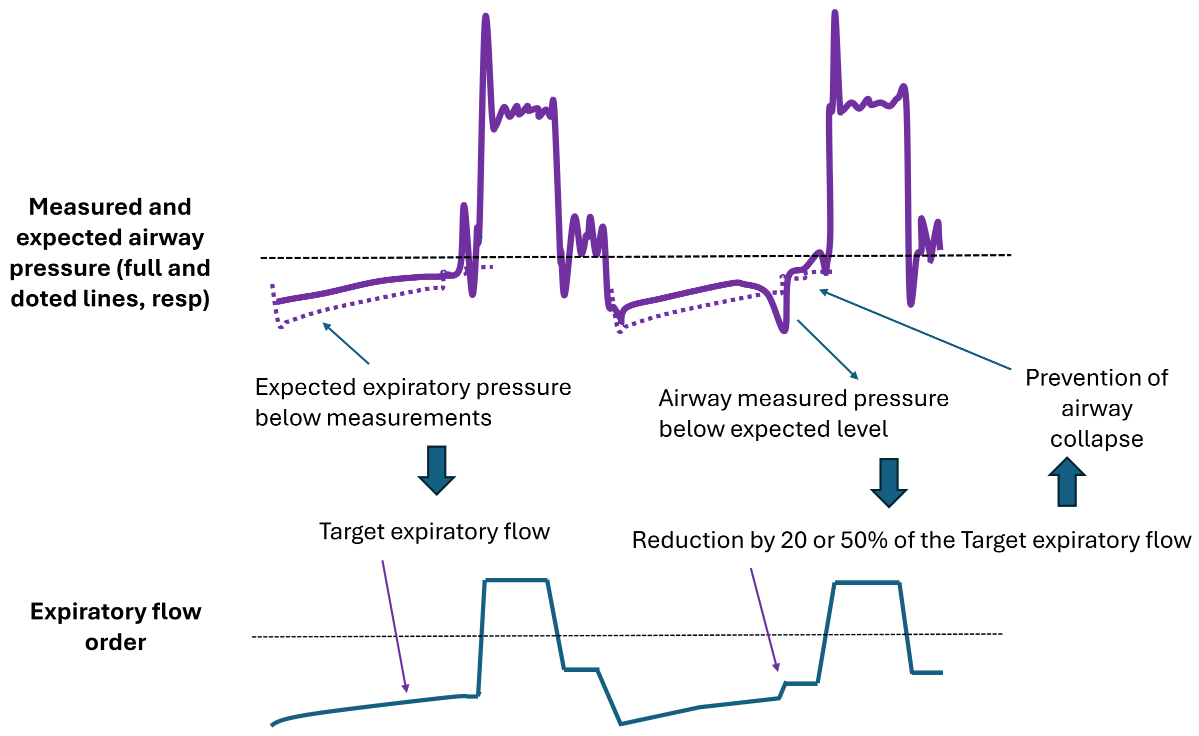

Supplemental Figure 4: Individual values of the hemodynamic and respiratory parameters at baseline and after oleic acid (OA) administration in the Control and Total Liquid Ventilation (TLV) groups

	Baseline	After OA administration
**PaO_2/FiO_2 ratio (mmHg), with FiO_2 = 100%**		
Control	572 ; 597 ; 573 ; 564 ; 625	38 ; 58 ; 80 ; 36 ; 79
TLV	574 ; 592 ; 551 ; 578 ; 587	47 ; 67 ; 97 ; 88 ; 58
**$PaCO_2$ (mmHg)**		
Control	44 ; 39 ; 48 ; 41 ; 40	87 ; 58 ; 62 ; 79 ; 33
TLV	44 ; 45 ; 45 ; 47 ; 48	76 ; 54 ; 59 ; 59 ; 69
**Respiratory compliance (ml/cmH2O/kg)**		
Control	1.1 ; 0.9 ; 1.0 ; 1.4 ; 0.7	0.3 ; 0.4 ; 0.3 ; 0.4 ; 0.3
TLV	1.0 ; 1.1 ; 0.7 ; 1.0 ; 1.0	0.3 ; 0.4 ; 0.3 ; 0.6 ; 0.4
**Heart rate, /min**		
Control	103 ; 70 ; 74 ; 74 ; 81	137 ; 196 ; 119 ; 157 ; 103
TLV	98 ; 66 ; 75 ; 124 ; 73	158 ; 120 ; 149 ; 148 ; 118
**Mean systemic blood pressure, mmHg**		
Control	87 ; 103 ; 99 ; 98 ; 68	48 ; 96 ; 84 ; 64 ; 82
TLV	115 ; 90 ; 86 ; 113 ; 92	95 ; 81 ; 88 ; 93 ; 78
**Mean pulmonary arterial pressure (mmHg)**		
Control	13 ; 17 ; 16 ; 19 ; 19	40 ; 43 ; 45 ; 48 ; 40
TLV	20 ; 18 ; 17 ; 18 ; 17	47 ; 42 ; 45 ; 34 ; 45
